# Fc receptor-like 2 (FCRL2) is a novel marker of low-risk CLL and refines prognostication based on *IGHV* mutation status

**DOI:** 10.1038/s41408-019-0207-7

**Published:** 2019-05-15

**Authors:** Lauren K. Shea, Kazuhito Honjo, David T. Redden, Edlue Tabengwa, Ran Li, Fu-Jun Li, Mikhail Shakhmatov, Nicholas Chiorazzi, Randall S. Davis

**Affiliations:** 1 Department of Medicine, University of Alabama at Birmingham, Birmingham, AL USA; 2 Department of Biostatistics, University of Alabama at Birmingham, Birmingham, AL USA; 3 Karches Center for Chronic Lymphocytic Leukemia Research, The Feinstein Institute for Medical Research, Northwell Health, Manhasset, NY USA; 4 Department of Microbiology, University of Alabama at Birmingham, Birmingham, AL USA; 5 Department of Biochemistry and Molecular Genetics, University of Alabama at Birmingham, Birmingham, AL USA; 6 Comprehensive Cancer Center, University of Alabama at Birmingham, Birmingham, AL USA

**Keywords:** Chronic lymphocytic leukaemia, Translational research, Prognosis

Chronic lymphocytic leukemia (CLL) is the most prevalent leukemia in Western countries and is characterized by a progressive accumulation of CD5^+^ monoclonal B cells in the blood and bone marrow. The clinical course in CLL is remarkably heterogeneous; the monoclonal population may expand in a protracted fashion over many years or increase rapidly, leading to bulky infiltration of lymphoid organs, progressive cellular and humoral immunodeficiency, autoimmunity, and cytopenias^1,2^. Prognostication at the time of diagnosis has important implications for patient care and has been an active area of investigation over the past several decades. Various means of risk stratification have been developed and validated, including clinical stage^3,4^, *IGHV* mutation status^5^, cytogenetic abnormalities^6^, and expression of surface proteins such as CD38^5,7^ and CD49d^8^. Composite prognostic indices, such as the German CLL Study Group prognostic score and the CLL-IPI, have also been proposed^9,10^. Despite an abundance of validated prognostic indicators, decisions to initiate treatment are still based on the presence of symptoms and/or cytopenias, and there is no evidence yet that preemptive treatment based on adverse prognostic factors improves overall survival (OS). Therefore, the search for reliable and widely available indicators that can be easily assayed at diagnosis and integrated to refine prognostication in CLL is ongoing.

Our own studies have focused on a family of Fc receptor-like (FCRL1-5) molecules that have tyrosine-based signaling potential and preferential expression by B lymphocytes^11^. In healthy donor peripheral blood mononuclear cell samples, the family member designated FCRL2 identifies subsets of CD19^+^CD27^+^ memory B cells and inhibits activation when co-ligated with the B-cell receptor, but has not been found to bind Ig^12^. Interestingly, in a series of 107 cryopreserved CLL samples, FCRL2 emerged as a powerful prognostic indicator^13^. FCRL2 expression was significantly elevated on CLL samples with mutated (M-CLL) as compared to unmutated (U-CLL) *IGHV* sequences, showed 94.4% concordance with *IGHV* mutation status, and was superior to nine other factors (including *IGHV* status) analyzed in predicting time to first treatment (TFT). Extended follow-up of our original 107-patient cohort showed that FCRL2 expression was predictive of OS as well as TFT (Fig. 1a). As expected, *IGHV* mutation status was also predictive of OS in our cohort. These encouraging results prompted us to pursue optimization of assaying FCRL2 expression to further its potential clinical use.

**Fig. 1:**
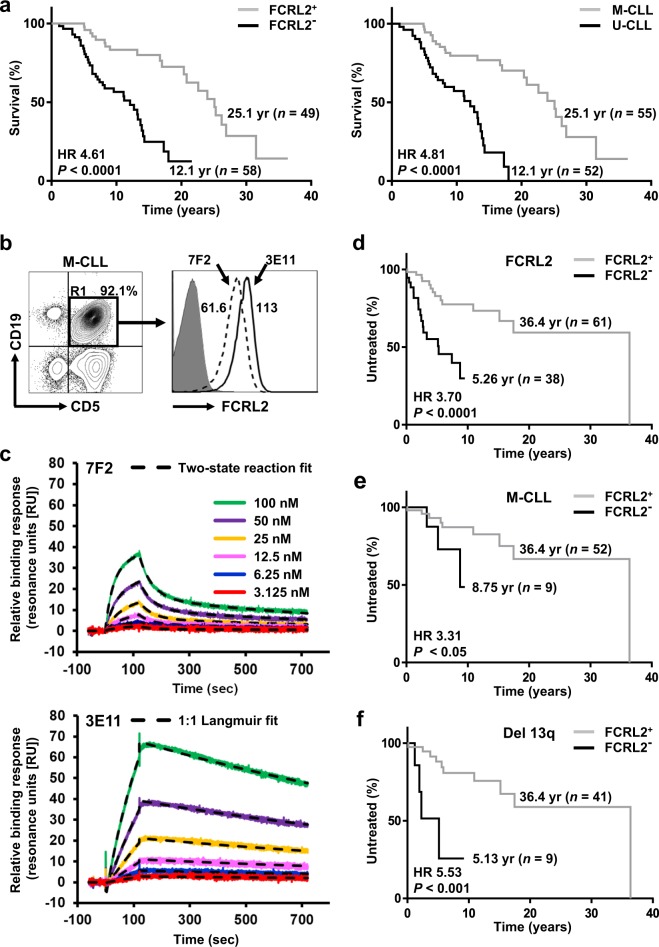
**a** FCRL2 expression predicts overall survival (OS) in CLL patients. Extended clinical follow-up of a previously characterized cohort of CLL patients (*n* = 107) assessed according to FCRL2 staining by flow cytometry using the 7F2 mAb as previously described^13^. Kaplan-Meier plots for OS of FCRL2-positive and negative patients were compared using the log-rank test, with Hazard Ratio (HR) and *P*-value displayed adjacent to the curves (left). Similar analysis was performed to compare OS for patients with mutated (M-CLL) and unmutated (U-CLL) *IGHV* (right). All statistical analysis was performed using SAS 9.1 (SAS Institute) and the RPART Package—R version 2.5.1 (The R Foundation for Statistical Computing). Graphs were generated using GraphPad Prism 7.00 software. **b** 3E11 shows strong and selective staining reactivity for FCRL2 on CLL cells by flow cytometry. PBMCs were freshly isolated using Lymphocyte Separation Medium (Mediatech) at the time of study enrollment and analyzed immediately. Cells were stained with anti-CD5-Briliant Violet (BV) 421 (clone UCHT2, BD Biosciences) and anti-CD19-allophycocyanin (APC) (clone HIB19, BD) to identify the expanded CLL population. Cells were then analyzed by flow cytometry after co-staining with the following anti-FCRL2 mAb clones: directly with PE-conjugated 3E11 or indirectly with biotinylated 7F2^13^ followed by streptavidin (SA)-PE (BD). After gating the lymphoid population according to typical light scatter characteristics, a total of 1−5 × 10^5^ events were acquired for analysis using an LSR flow cytometer (BD) and analyzed with FlowJo software (Tree Star). Histograms for FCRL2 are derived from the gated CD19^+^CD5^+^ CLL population (R1) and the subset frequency is noted. The numbers in the histogram indicate the respective mean florescence intensity ratio (MFIR) as determined by dividing the MFI of the antigen-specific fluorochrome-conjugated mAb by the MFI of the irrelevant fluorochrome-conjugated isotype-matched negative control mAb. **c** 3E11 shows stronger binding kinetics with FCRL2 by surface plasmon resonance (SPR). SPR analysis was used to determine the kinetics of 7F2 (above) or 3E11 (below) binding to an FCRL2-Fc recombinant protein generated as previously described^15^. SPR analysis was performed using a Biacore T100 instrument upgraded to T200 specifications equipped with a CM5 sensor chip (GE Healthcare). Standard amine-coupling chemistry was used to immobilize approximately 10,000–10,500 RU of a rat anti-mouse IgG1κ capture Ab (clone 187.1, Southern Biotech) onto the CM5 chip as recommended by the manufacturer. For the subsequent capture step, the 3E11 and 7F2 mAbs were diluted to 2 µg/ml in HBS-EP+(0.01 M HEPES, pH 7.4, 0.15 M NaCl, 3 mM EDTA, 0.05% vol/vol Surfactant P20 [GE]) running buffer and each mAb was injected over the 2nd or 4th surfaces for 60 s at a flow rate of 10 μl/min at 25 °C. No mAb was injected over the 1st or 3rd channels, which served as reference surfaces. For measuring kinetics, two-fold serial dilutions of FCRL2-Fc or Fc-only control proteins (in HBS-EP+) ranging from 100–3.125 nM, as well as blank buffer (for baseline subtraction), were sequentially injected over the rat anti-mouse IgG1κ only (reference) or the captured 3E11 or 7F2 surfaces at 30 µl/min at 25 °C. Binding interactions were monitored for 2 min association and 10 min dissociation periods. Prior to curve fitting, specific real-time binding sensorgrams were obtained by using the standard double-referencing procedure, whereby the control surface and buffer blank injections are subtracted from the binding data. Biacore T100 Curve Fit Evaluation Software Version 2.0.3 (GE) was used to determine binding kinetics. The binding curves from six concentrations of FCRL2-Fc in the two-fold dilution series were then globally fit into a typical 1:1 Langmuir binding model or into a two-step model with mass transport limitation to determine the association (*ka*) and dissociation (*kd*) rate constants as well as the equilibrium dissociation constant (*KD*) of each interaction (*KD* *=* *kd/ka*). Representative sensorgrams (colored lines) and kinetic model fits (black dashed-lines) are shown. A control Fc-only protein injected over the 7F2 and 3E11 surfaces did not show detectable binding to either mAb (not shown). For 7F2 (above), *ka*_*1*_ = 1.85 × 10^5^ (1/Ms), *kd*_*1*_ = 1.79 × 10^−2^ (1/s), *ka*_*2*_ = 3.93 × 10^−3^ (1/Ms), *kd*_*2*_ = 1.32 × 10^−3^ (1/s), and *KD* = 2.43 × 10^−8^ (M). For 3E11 (below), *ka* = 5.54 × 10^4^ (1/Ms), *kd* *=* 5.58 × 10^−4^ (1/s), and *KD* = 1.06 × 10^−8^ (M). **d** FCRL2 staining with 3E11-PE is predictive of TFT in CLL patients. The 3E11 anti-FCRL2 mAb was used to analyze samples from a new cohort of CLL patients (*n* *=* 99). Staining of freshly isolated PBMCs from CLL samples was performed as in (**b**). CLL samples with ≥76.8% positivity for FCRL2 were designated FCRL2-positive and those with <76.8% positivity were designated FCRL2-negative as described in the text. The Kaplan-Meier plot represents TFT for FCRL2-positive and negative patients and was generated and analyzed as in (**a**). **e**, **f** FCRL2 staining refines prognostication in patients with low-risk CLL. Kaplan-Meier plots stratified by FCRL2 expression demonstrate the median TFT for patients with M-CLL (**e**) and 13q deletion alone (**f**). Kaplan-Meier plots represent TFT for FCRL2-positive and negative patients and were generated and analyzed as in Figure **a**

We first set out to develop an FCRL2-specific monoclonal antibody (mAb) with superior reactivity and binding properties relative to our prior clone 7F2^13^. After institutional IACUC approval, BALB/c mice were hyper-immunized with BW5147 mouse T cell line retroviral transductants expressing FCRL2. Two separate fusions were performed and an extensive analysis of 92 FCRL2-reactive hybridoma clones was undertaken. This screen led to the identification of 3E11, a mouse IgG1κ subclone that does not cross-react with the five other FCRL molecules expressed by human lymphocytes (Fig. S1). Blocking experiments indicated that 7F2 and 3E11 bind distinct epitopes on FCRL2 (Fig. S2). To pursue further characterization of the 3E11 clone, we directly conjugated it with phycoerythrin (PE) and stained PBMCs from CLL donors to assess its reactivity. The direct conjugation of 3E11 with PE did not interfere with binding to CLL cells (data not shown). Analysis of a M-CLL sample stained indirectly with the previously described anti-FCRL2-mAb 7F2 (biotinylated and co-stained with SA-PE) versus directly with 3E11-PE revealed stronger MFI reactivity for the latter mAb (Fig. 1b). As expected, the staining intensity of individual CLL samples for 3E11 and 7F2 showed a strong linear correlation (*R*^2^ = 0.93) (Fig. S3). We also compared the kinetics of the two mAbs by examining their relative interactions with an FCRL2-Fc chimeric protein by surface plasmon resonance (SPR). 3E11 had a higher overall nanomolar affinity (approximately 2.3-fold) for the FCRL2 recombinant protein, as well as a slower dissociation rate than 7F2 (Fig. 1c). In addition, while 3E11 binding resembled a typical 1:1 Langmuir model, the 7F2 kinetic data indicated that its epitope interaction is more complex and resembled a two-step reaction. These data demonstrated that compared to 7F2, the 3E11 mAb exhibits higher surface staining reactivity, 1:1 binding kinetics, and stronger overall affinity for FCRL2.

We next employed the PE-conjugated 3E11 mAb to validate the prognostic significance of FCRL2 by staining samples from a new cohort of CLL patients. Out of 182 consecutive patients with CLL (as defined by NCI criteria)^14^ seen at our institution and consented with IRB approval for banking over an approximately four-year period (May 2014 through February 2018), we obtained complete clinical and molecular data on 99 patients. Characteristics of this new patient cohort are shown in Table 1. We used maximum Youden Index values to determine the optimal threshold cutoff value for percent positivity of 3E11 based on the Receiver-Operating Characteristic (ROC), in order to maximize correlation with *IGHV* mutation status. This analysis identified 76.8% as the optimal cut-off value. CLL samples with ≥76.8% reactivity were designated FCRL2-positive and those with <76.8% reactivity were designated FCRL2-negative. Consistent with its close association with *IGHV* mutation status and our prior results obtained using the 7F2 mAb^13^, FCRL2 was also a powerful predictor of TFT in the new cohort, with median TFT for FCRL2-positive cases 36.4 years as compared to 5.26 years for FCRL2-negative cases (HR 3.70, 95%CI 1.64 – 8.38, *P* < 0.0001, Fig. 1d). *IGHV* status was also predictive of TFT, with median TFT for M-CLL patients 36.4 years as compared to 2.76 years for U-CLL patients (HR 6.28, 95%CI 2.61–15.1, *P* < 0.0001), and both FCRL2 and *IGHV* status were superior predictors relative to CD38 (data not shown). Importantly, among patients with M-CLL, FCRL2 could further refine prognostic capability. The combination of M-CLL and positive FCRL2 surface expression conveyed a superior prognosis. Median TFT was 36.4 years for FCRL2-positive patients with M-CLL as compared to only 8.75 years for FCRL2-negative patients with M-CLL (HR 3.31, 95%CI 0.465–23.6, *P* < 0.05, Fig. 1e). However, among patients with U-CLL, FCRL2 did not improve prognostication of TFT (data not shown). Similarly, for those with 13q deletion alone, FCRL2 was able to stratify patients into lower (median TFT 36.4 years) or higher (median TFT 5.13 years) risk groups (HR 5.53, 95%CI 0.685–44.6, *P* < 0.001, Fig. 1f).

**Table 1:** Clinical characteristics of CLL samples

Parameter	All patients	M-CLL	U-CLL
No. of patients (%)	99	61 (61.6)	38 (38.4)
Sex, no. (%)			
Male	53 (53.5)	28 (45.9)	25 (65.8)
Female	46 (46.5)	33 (54.1)	13 (34.2)
Age at diagnosis, y			
Median	62	59	64
Range	40–84	40–84	40–80
Rai stage, no (%)			
0	78 (78.8)	55 (90.2)	23 (60.5)
I-II	16 (16.2)	5 (8.2)	11 (28.9)
III-IV	5 (5.1)	1 (1.6)	4 (10.5)
Time to treatment			
Median time to first treatment, y	2.8	5.7	2.3
No. treated (%)	31 (31.3)	12 (19.7)	19 (50)
No. censored (%)	68 (68.7)	49 (80.3)	19 (50)
Cytogenetics			
17p deletion, no. (%)	8 (8.1)	2 (3.3)	6 (15.8)
11q deletion, no. (%)	7 (7.1)	0(0)	7 (18.4)
13q deletion, no. (%)	50 (50.5)	44 (72.1)	6 (15.8)
12q trisomy, no. (%)	15 (15.2)	3 (4.9)	12 (31.6)
Normal, no. (%)	19 (19.2)	12 (19.7)	7 (18.4)
FCRL2 expression			
Positive, no. (%)	61 (61.6)	52 (85.2)	9 (23.7)
Negative, no. (%)	38 (38.4)	9 (14.8)	29 (76.3)
CD38 expression			
Positive, no. (%)	13 (13.1)	1 (1.6)	12 (31.6)
Negative, no. (%)	86 (86.9)	60 (98.4)	26 (68.4)

Samples were stained for FCRL2 expression as described in Fig. 1b. Median time from diagnosis to sample collection and flow cytometry was 4.29 years (range 0–34.2 years). CD38 surface measurements were determined with anti-CD38-PE (clone HB-7, BD) according to published criteria^5,7^. A cutoff value of >30% was used to determine a positive result. FISH for trisomy 12q, 13q deletion, 11q deletion, and 17p deletion was performed according to standard methodology by the UAB Department of Genetics. Cytogenetic profiles were categorized according to the original hierarchy as described^6^. *IGHV* mutation status for CLL samples was either determined in-house according to established methods as previously described^13^ or performed by the Mayo Clinic Laboratories (Rochester, MN). FISH and *IGHV* sequencing were determined at the time of diagnosis for the majority of cases

We next performed a multivariable Cox proportional hazard forward selection model including FCRL2, CD38, *IGHV* mutation status, and cytogenetics (trisomy 12q, 13q deletion, normal, 11q deletion, and 17p deletion). The factors that emerged as the best predictors of TFT were *IGHV* status and trisomy 12q. Likely due to its co-linearity with *IGHV*, FCRL2 expression did not enhance prognostic power when added to this model. However, when *IGHV* status was excluded, FCRL2 expression and 17p deletion emerged as the most powerful prognostic factors (Table S1).

Though not superior to *IGHV* status in predicting TFT in our patient cohort, FCRL2 expression was able to further refine and extend prognostication in M-CLL patients with an already favorable prognosis. This is certainly important to individual patients, who may suffer from significant emotional distress upon being diagnosed with a malignancy that is largely incurable, and then being informed that observation until progression is the standard of care for asymptomatic individuals. A positive prognostic marker such as FCRL2, readily testable by flow cytometry, may provide reassurance to patients and help clinicians determine frequency of follow-up, though this should be evaluated in clinical trials. In addition, we are hopeful that investigation of the functional significance of FCRL2 expression levels in normal and malignant B cells may provide further insight into the biology of CLL

There are several caveats to our data that should be acknowledged. First, confirmation of these results should be pursued with an independent validation cohort of CLL samples. The retrospective nature of this study, with some patients diagnosed more than thirty years prior to data analysis, made obtaining complete data from the time of diagnosis challenging and thus prevented calculation of composite prognostic indices such as the CLL IPI^10^. Furthermore, many of our patients underwent first treatment prior to the development of the molecular targeted agents that are now transforming the treatment landscape in CLL, and further study will be needed to examine the role of FCRL2 in prognostication (and possibly prediction of response) as CLL therapy evolves. Nevertheless, the fact that FCRL2 was able to refine prognostication of patients with an already excellent prognosis (M-CLL, deletion 13q positive) indicates a need for further prospective studies of this readily assayable marker in both prognostication and prediction of response.

In summary, we have shown that FCRL2 is a powerful predictor of both TFT and OS in CLL and have validated the prognostic significance of FCRL2 in a new cohort of patients. We have developed a novel PE-conjugated mAb to FCRL2 that produces robust and specific staining of clinical samples, with implications for refining and extending prognostication of low-risk disease. We are hopeful that these findings will enhance both research and clinical care in CLL.

## Supplementary information

Supplemental Figures

Supplemental Figure Legends for S1, S2, and S3 and Table SI
