# Supplementary figures and images for: Fc receptor-like 2 (FCRL2) is a novel marker of low-risk CLL and refines prognostication based on IGHV mutation status
Source: Blood Cancer J. 2019 May 15;9(6):47. doi: 10.1038/s41408-019-0207-7 (PMC6520396; doi:10.1038/s41408-019-0207-7)

**Figure S1**

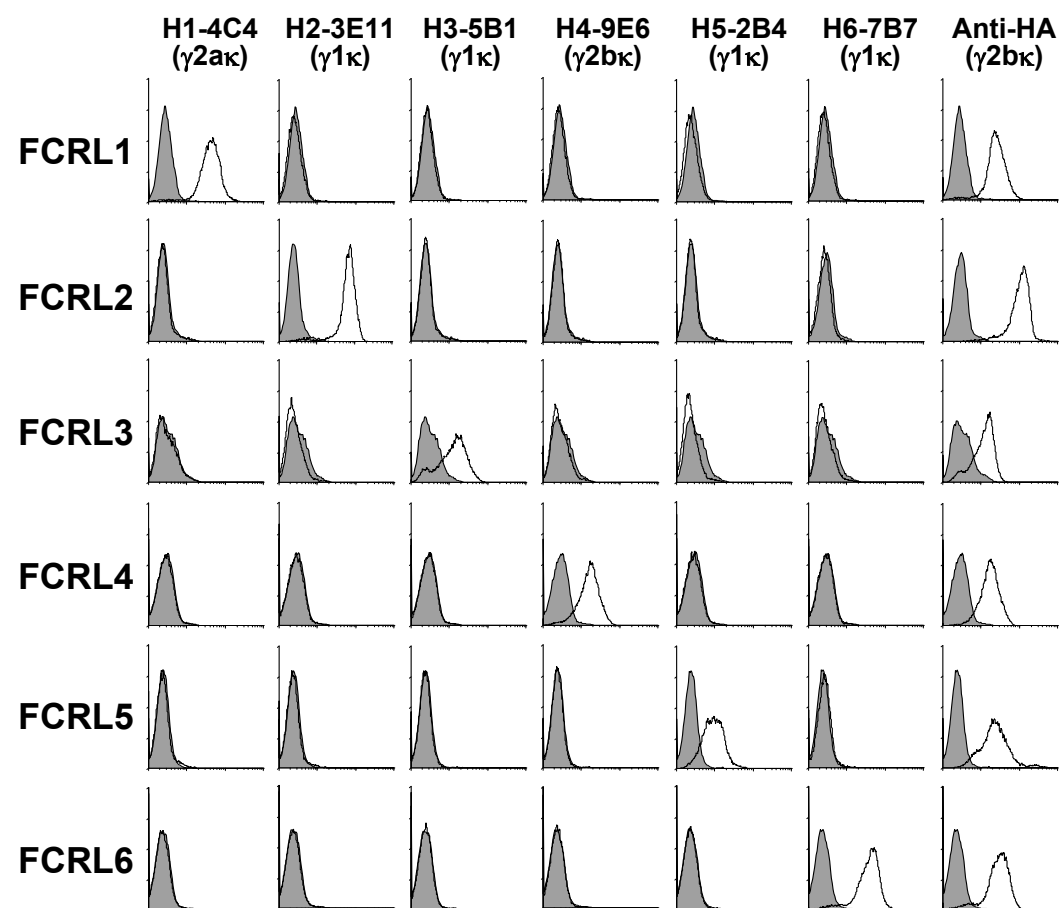

**Figure S2**

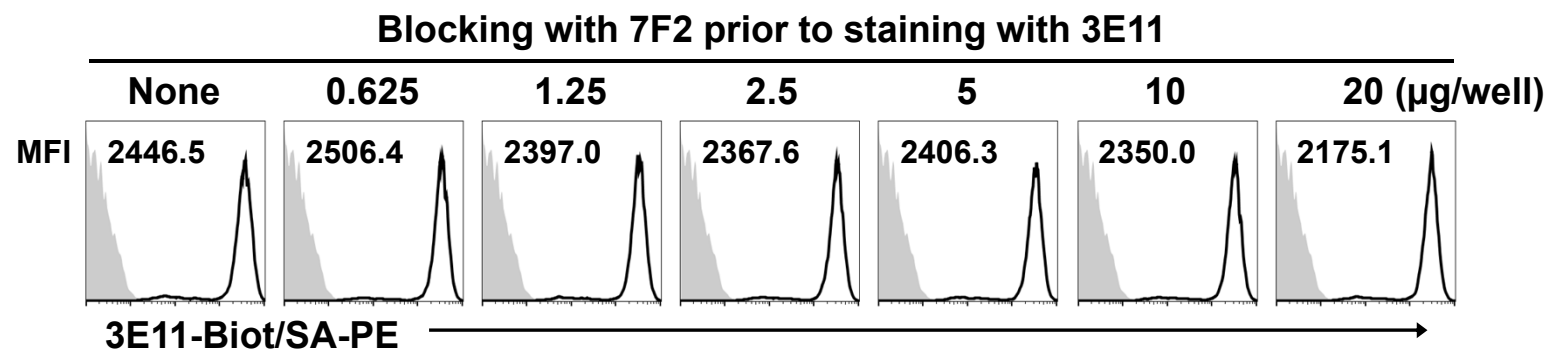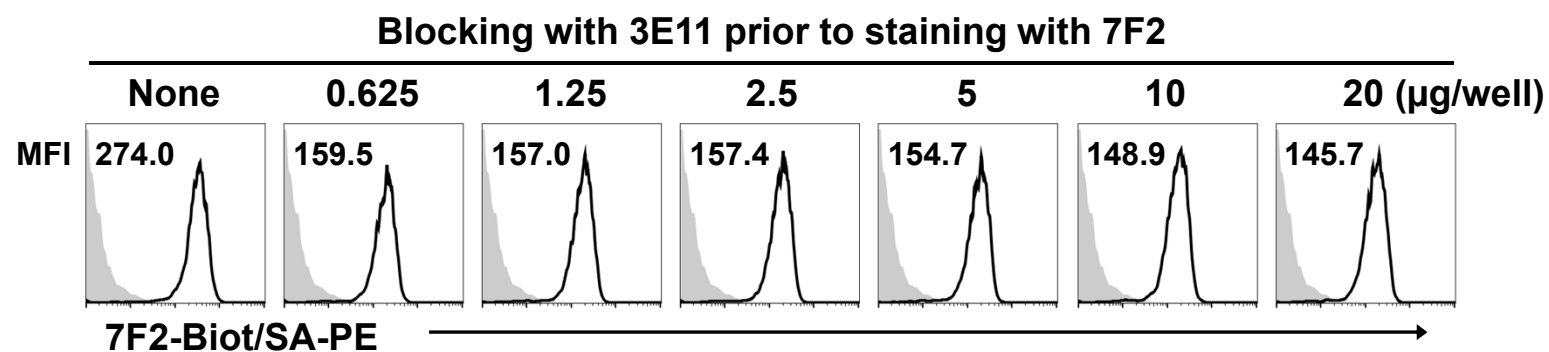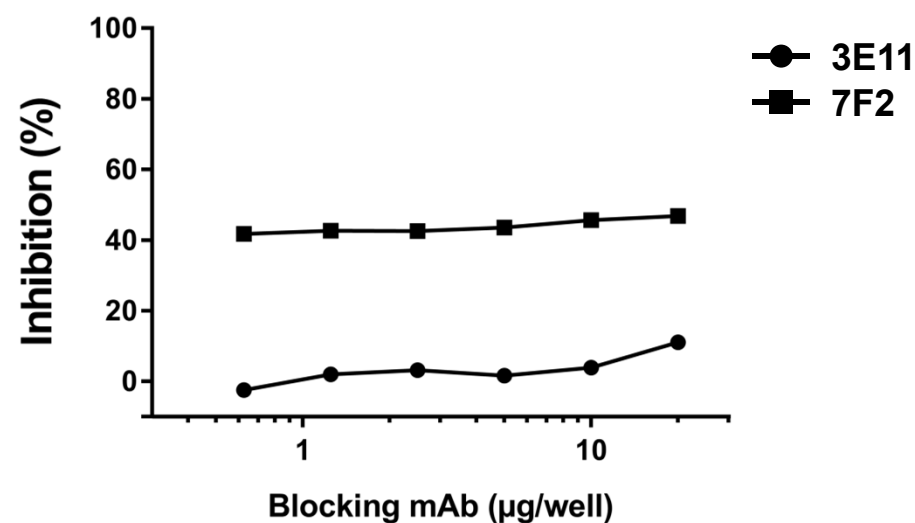

Figure S3

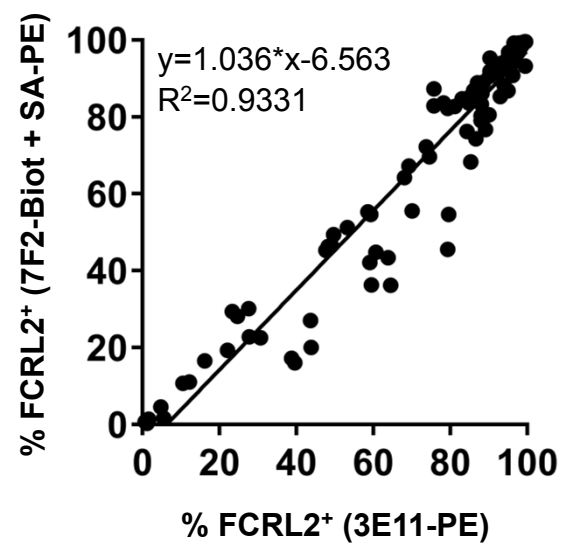

Supplement: Supplementary file 1 — Supplemental Figures [file 41408_2019_207_MOESM1_ESM.pdf]
