# Supplementary material for: Fc receptor-like 2 (FCRL2) is a novel marker of low-risk CLL and refines prognostication based on IGHV mutation status
Source: Blood Cancer J. 2019 May 15;9(6):47. doi: 10.1038/s41408-019-0207-7 (PMC6520396; doi:10.1038/s41408-019-0207-7)
Supplement: Supplementary file 2 — Supplemental Figure Legends for S1, S2, and S3 and Table SI [file 41408_2019_207_MOESM2_ESM.docx]

**Supplemental Figure Legends**

**Figure S1. The 3E11 mAb is FCRL2-specific.**

BW5147 mouse T cell line retroviral transductants (0.5 x 10^6^) expressing N-terminal HA-tagged FCRL1-6 proteins were stained with the indicated mAbs (respective mouse isotypes are shown), or an isotype-matched control, followed by counter-staining with secondary polyclonal goat anti-mouse Ig-PE Abs (Southern Biotech) before flow cytometry analysis. Positive control staining was performed with an anti-HA mAb (clone 12CA5, Roche). Note that FCRL6, which is expressed by T and NK cells, was also included for analysis.

**Figure S2. The 3E11 and 7F2 mAbs bind distinct epitopes on FCRL2.**

BW5147 T cell line transductants expressing FCRL2 (0.5 x 10^6^) were first blocked with increasing concentrations of either unlabeled 7F2 or 3E11 mAbs, washed, and then stained with 1 µg of biotinylated 3E11 or 7F2, respectively. Staining reactivity of the biotinylated mAbs was detected with SA-PE. Unblocked controls (none) were stained solely with biotinylated mAbs and SA-PE. Inhibition was calculated by dividing the MFI of FCRL2 staining in blocked samples by the MFI of the unblocked control. Results (% inhibition) were expressed by subtracting the divided MFI value from 100%.

**Figure S3. The staining intensity of 3E11 and 7F2 on individual CLL samples shows a strong linear correlation.** The frequency of CLL cells positive for 3E11-PE versus 7F2-biotin followed by SA-PE was plotted (*n* = 99) to validate their respective staining on the same sample. The correlation coefficient is indicated in the plot.

|  | **Parameter** | **Hazard Ratio** | **95%CI** | ***P* value** |
| --- | --- | --- | --- | --- |
| **Including *IGHV*** |  |  |  |  |
|  | *IGHV* status | 0.062 | 0.03-0.16 | < 0.0001 |
|  | 12q trisomy | 0.23 | 0.08-0.71 | < 0.05 |
| **Excluding *IGHV*** |  |  |  |  |
|  | FCRL2 | 0.25 | 0.11-0.56 | < 0.001 |
|  | 17p deletion | 2.9 | 1.09-7.89 | < 0.05 |

**Table SI.** **FCRL2 and high-risk cytogenetics predict TFT in multivariate analysis when *IGHV* status is excluded.** The Cox model of multivariate analysis with stepwise selection was used to determine the factors most highly predictive of TFT. The following factors were included in analysis: FCRL2, CD38, *IGHV* mutation status, and cytogenetics (trisomy 12q, 13q deletion, normal, 11q deletion, and 17p deletion). All statistical analysis was performed using SAS 9.1 (SAS Institute) and the RPART Package - R version 2.5.1 (The R Foundation for Statistical Computing).
